# Kallikrein 3 and vitamin D receptor polymorphisms: potentials environmental risk factors for prostate cancer

**DOI:** 10.1186/1746-1596-9-84

**Published:** 2014-04-22

**Authors:** Jianpeng Hu, Zhen Qiu, Liansheng Zhang, Feilun Cui

**Affiliations:** 1 Department of Urology, People’s Hospital Affiliated of Jiangsu University, Zhenjiang 212002, Jiangsu, China

**Keywords:** Prostate cancer, Nucleotide polymorphisms, Environmental risk factor

## Abstract

**Objective:**

To investigate the relationship and interaction of the single nucleotide polymorphisms (SNPs) of KLK3 and VDR and environmental factors with the predisposition to prostate cancer within Chinese population.

**Methods:**

The comparison between 108 patients and 242 healthy people was carried out by using the TaqMan/MGB Probe Technology to determine the genotypes of KLK3(rs2735839 is located between KLK2 and KLK3) and VDR (rs731236 is located exon 9). Univariate and multivariate logistic regression model were used to assess the connection of genetic polymorphisms and environmental risk factors with PCa by collecting demographic information, as well as BMI, consumption of cigarettes, alcohol, and tea, exercise, and other environmental risk factors.

**Results:**

The appearing frequencies of AA, AG, and GG genotypes at the SNPs rs2735839 (A/G) for KLK3 were 13.89%, 62.96% and 23.15% in PCa and 37.19%, 44.63%, 18.18% in control, respectively; these two groups are statistically different (P = 0.00). While the appearing frequencies of TT, TC, and CC genotypes at the SNPs rs731236 (T/C) for VDR were 88.89%, 9, 26%, 1.85% and 90.50%, 9.10%, 0.40% in control, respectively, with no significant statistical difference between the two group. The study confirmed decreasing risk in tea drinkers (OR = 0.58, 95% CI = 0.35-0.96).

**Conclusions:**

Our studies indicate that environmental factor-tea drinking is associated with the development of PCa. The habit of drinking tea is a protective factor against PCa. The SNPs rs2735839 for KLK3 is strongly related to the development of PCa, while the SNPs rs731236 for VDR is not.

**Virtual slides:**

The virtual slide(s) for this article can be found here: http://www.diagnosticpathology.diagnomx.eu/vs/9759981571058803.

## 

The occurance of prostate cancer (PCa) in Europe and America is higher than other area. PCa is ranked the second most commonly occuring cancer among the male cancer patients in Europe and American, the major cause of male deaths in developed countries [1]. With the aging of the population and the improvement of living conditions in recent years, PCa incidence among male Chinese has been increasing every year, China’s evarage standardized incidence rate was 1.6/10 million in 2002, while in Shanghai in 2000, the standardized incidence rate was 7.7 / 10 million. It is estimated that by 2010 in Shanghai, the incidence rate may be more than 10/100 000 [2]. PCa is a common multifactorial, polygenic disease with significant heterogeneity. The mechanism of its inheritance and pathogenesis is very complex; Environmental factors and genetic factors jointly contribute to the occurrence of PCa. The studies by Sonoda et al [3] suggest that environmental factors such as diet and lifestyle, play an important role in the pathogenesis of PCa. Loci genome-wide association studies of multiple ethnic groups in recent years has identified some susceptibilities associated with PCa, however, the majority research was carried out among African and European ethnic groups [4-6], while related research on the Chinese population has been very limited.

The kallikrein 3 (Kallikrein 3, KLK3) gene is located on chromosome 19q13.33, which encodes prostate specific antigen (PSA) and is a serine protease kallikrein family member. Like other kallikrein, PCa kallikrein may be involved the occurrence of PCa and metastasis [7,8]. The vitamin D receptor (Vitamin D Receptor, VDR) gene is located on chromosome 12q13.11, belonging to the steroid hormone receptor superfamily. About the mechanism of the VDR gene polymorphism and cancer relationship, it is now widely recognized that 1, 25 (OH) 2D3 and its analogs fix tumor cell growth cycle in the G1 phase via VDR, thereby inhibiting tumor cell proliferation. VDR gene polymorphism essentially unchanged the order of amino acids in VDR proteins, but affects the VDR protein expression at the transcriptional level. Change of the VDR gene polymorphism was considered to be associated with PCa predisposition [9] or progressive genotype [10], but different opinions are reported [11].

It has been confirm that the single nucleotide polymorphisms (SNPs) rs2735839 of KLK3 and the SNPs rs731236 of VDR are associated with PCa. Seldom research has been on the realationship of those two SNPs and PCa occurrence within the Chinese ethic group. This study is focused on the relationship between the KLK3 and VDR gene SNPs as well as environmental risk factors, and the occurance of PCa within the Chinese population.

## Data and methods

### Patients

Clinical studies of the 108 PCa patients were carried out from October 2007 to April 2010 at The First Affiliated People’s Hospital of Jiangsu University. These patients were confirmed to have PCa, with methods including B ultrasound, CT check and pathological diagnosis. The control group of 242 healthy people are from the community population without any history of PCa with normal serum PSA level.

### Methods

#### Epidemiological investigation

Investigators developed a systematic questionnaire, and the survey was implemented via interiews of the patients. All the questionnaires were truthfully filled out with the consent of the respondents. Environmental risk factors, including body mass index (BMI), comsumption of alcohol, tea, and cigarettes, exercise, are evaluated. BMI factor is classified into two groups: BMI ≤ 24 kg/m2 and BMI > 24 kg/m2; regular alcohol consumption is defined as more than once every week and continued for at least 6 months. Tea consumption: seldom or never consumption (<4 times/week); regular tea comsumption (≥ 4 times/week); Smoking: non-smoker, moderate smoker (≤ 20 cigarettes/day), heavy smoker (> 20/day); exercise: seldom exercise (<4 times/week), regular exercise ( ≥ 4 times/week); each exercise needs to last for as least half an hour.

#### DNA extraction

3 ml peripheral venous blood was extracted from each patient. After anti-coagulation by EDTA, peripheral blood genomic DNA was extracted using the extraction kit produced by Beijing TIANGEN, and stored at -20°C.

#### KLK3 and VDR genotype analysis

Primers and probes (Table 1) are desinged by Jikang Biological Science and Technology Co., Ltd. The total volume of the PCR reaction system is 10 μl, including 5 μl Ex TaqTM Hot Start Version (TaKaRa), 0.2 μl primer, 0.3 μl probe, 3 μl deionized water, and 1 μl DNA template. The reaction was carried out on a real-time quantitative PCR instrument (LightCycle 480) and the paramters are set as the following: 50°C for 2 min, 95°C for 10 min, followed by 95°C for 30s and 58°C for 1 min. 40 cycles in total were performed. Fluorescence signal was collected and automatically analyzed to identify the SNP genotype.

**Table 1:** Primers and probes for genotyping of KLK3 and VDR SNPs

**Gen**	**rs**	**Varian**	**Primer probe**
KLK	2725839	A/G	Forward: TCCTCAACCTTCCCTATTTCTG
			Reverse: GTGAGGGAAAGGGAGAAGATGA
			FAM-CATGGTCCATTGGCCACAAGAC-TAMRA
			HEX-CATGGTCCACTTGGCCACAGACA--TAMRA
VDR	731236	T/C	Forward: CGTGCCCACAGATCGTCC
			Reverse: TGTACGTCTGCAGTGTGTTGGA
			FAM-CCGCGCTGATTGAGGCCATC-TAMRA
			HEX-CGCGCTGATCGAGGCCATC-TAMRA

KLK: Kallikren 3; VDR: Vitamin D receptor; SNPs: single nucleotide polymorphisms.

### Statistical analysis

Both laboratory testing results and epidemiological data were entered into a computer, compared and checked to ensure the accuracy of the entry. Hardy-Weinberg (HWE) Genetic equilibrium analysis was carried out on the object of the study; SPSS16.0 statistical analysis was applied: χ2 test analysis of genotype frequencies, odds ratio (odds ratio, OR) and 95% CI estimate on the correlation strength of each studied factor with PCa; Logistic regression estimates on the relationship of the independent risk genotypes and the disease; multiplying and additive model analysis, which sums the interaction analysis using the relative excess risk due to interaction (RERI) and 95% CI assessment of environmental risk factors and genetic polymorphisms interaction. The test level (α) was 0.05.

## Results

### The environmental risk factors

The patients were between age 51 an 88 years, with an average of (70.01 ± 7.26) years old; Jewett clinical stage: 32 cases, 28 cases of B stage, 32 cases of C stage, and 48 cases of D stage; Gleason score: 4 to 7 points for 76 cases, and 8 to 10 points for 32 cases. The age of the control group is between 49 and 86 years, with an average of (70.52 ± 7.11) years old. The analysis on the environmental factor-dependance of PCa occurance confirmed that alcohol consumption (48.15% vs 36.36%, P = 0.04), and tea consumption (62.96% vs 74.38%, P = 0.00); they are statistically different between the two groups. The estimated relative risk values OR are: 1.63 [95% CI (1.03 ~ 2.57)], 0.59 [95% CI (0.36 ~ 0.95)]. The details are confirm in Table 2.

**Table 2:** Environment factors of prostate cancer

**Characteristics**	**PCa**	**Control**	***OR *(*95% CI*)**	***P***
	**(*N*=108)**	**(*N*=242)**		
Age (Year)	70.01±7.26	70.52±7.11		.73
BMI(^∎^m^-2^)	24.93±4.06	23.91±2.74		0.11
Alcohol drinking				0.04
No	56(51.85)	154(63.64)	1.00	
Yes	*52*(48.15)	88(36.36)	1.63(1.03-2.57)	
Tea *n *(%)				0.00
Yes	68(62.96)	180(74.38)	1.00	
Smoking *n* (%)				
Never	51(47.22)	124(51.24)	1.00	
1-20 pieces/d	51(47.22)	92(38.02)	1.35(0.84-221.45)	
Sports *n* (%)				0.39
Yes	63(58.33)	156(63.22)	1.00	
No	45(41.67)	89(36.78)	1.23(0.77-1.95)	

BMI body mass index.

#### The analysis on the correlation of KLK3 and VDR genotype and PCa

**Figure 1:**
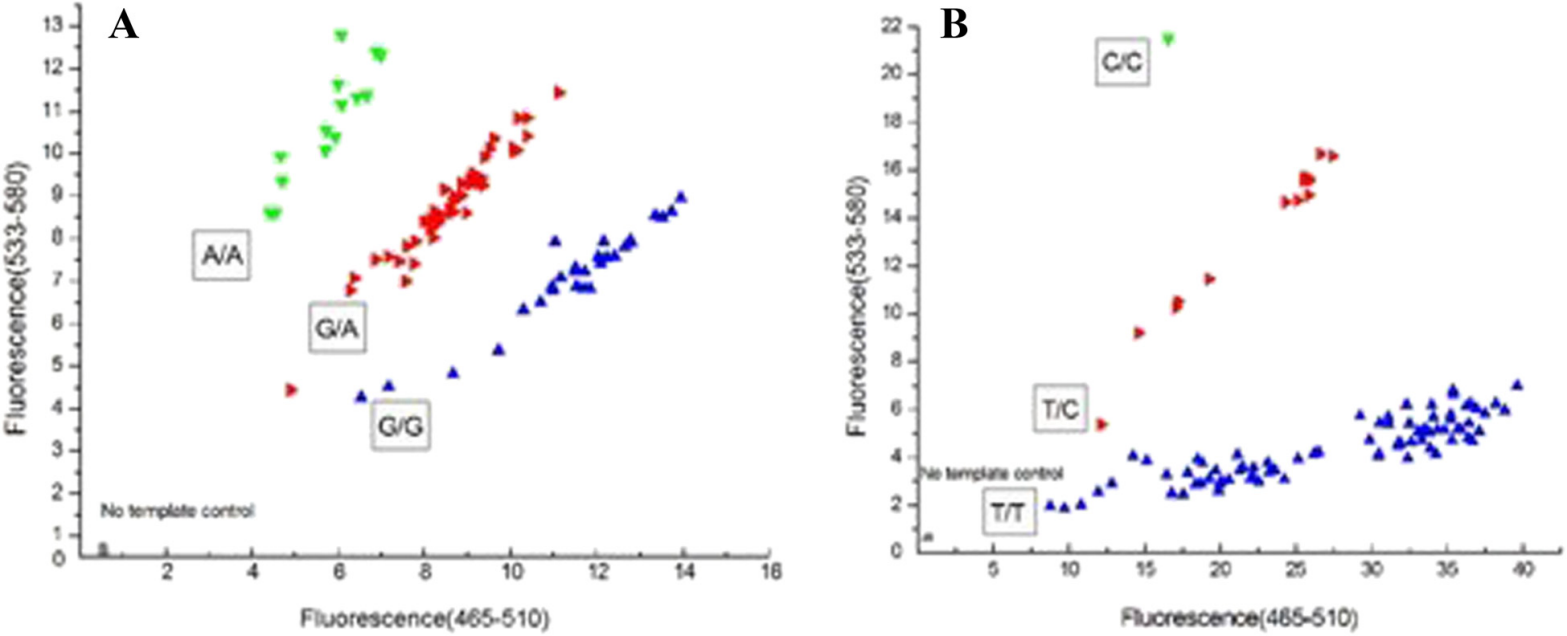
Real-time PCR genotyping of rs2735839 (A) and rs731236 (B).

The SNPs of KLK3 and VDR, rs2735899 and rs731236, genotyping results are confirm in Figure 1. The genotype distribution of KLK3 and a VDR from the control group is in Hardy-Weinberg equilibrium, and the P values are 0.25 and 0.58, respectively. As confirm in Table 3: the differences in genotype of the SNPs of KLK3, rs2735839, between the PCa patinets and the control groups was statistically significant (χ2 = 19.44, P = 0.00). GG or AG alleles, compared with AA, significantly increase the incidence of PCa (OR = 3.67, 95% CI, 2.01 - .72); VDR SNPs rs731236 genotype differences between the case and control groups was not statistically significant (χ2 = 1.65, P = 0.44).

**Table 3:** Two genotype frequencies of prostate cancer between Pca and control group

**SNP**	**PCA(N=108)**	**Controll (N=242)**	**OR (95% CI)**	***n*%**
rs2735839				
AA	15(13 89)	90(37.19)	1.0 (reference)	
AG	68(62.96)	108(44.63)	3.78(2.02)-7.06	
GG	25(23.15)	44(18.18)	3.41(1.64-7.11)	
GG+AG	93 (86.11)	152(62.81)	3.679(2.01-6.72)	
rs731236				
TT	96(88.89)	219(90.50)	1.0(reference)	
TC	10(9.26)	22(9.10)	1.04(0.47-2.27)	
CC	2(1.85)	1(0.40)	456(0.41-50.92)	
CC+CT	12(11.11)	23(9.51)	1.19(0.57-2.49)	

PCa: Prostate cancer; SNP: single nucleotide polymorphism; a 95% Coincidence interval.

2.2.2 The correlation between clinico-pathological characteristics and the two SNPs. rs2735839 (KLK3 gene) and rs731236 (VDR gene) of wild-type and heterozygous/mutation distribution with age, Gleason score and PSA level is not associated with PCa.

The frequency of SNPs rs2735839 carrying dangerous G allele was significantly higher than in the localized PCa than advanced PCa patients (P = 0.03), while the difference in the frequency of SNPs rs731236 carrying the risk allele C in localized PCa and advanced PCa patients is not statistically significant (P = 0.66), as confirm in Table 4.

**Table 4:** Two SNPs and clinicopathological characteristics in prostate cancer

**Variable**	**rs2735839**			**rs731236**		
	**AA**	**AG/GG**	***P***	**TTg**	**CC/CT**	***P***
Age (Year)			0.96			0.07
<70	7	44		51	3	
>70	8	49		45	9	
TNM classification			0.03			0.62
Localized	9	78		74	9	
Aggressive	6	15		22	3	
Gleason score			0.74			0.71
<7	10	66		67	9	
>7	5	27		29	3	
PSA *ρ*/ng^∎^ml^-1^			0.10			0.60
<10	2	32		31		
>	13	61		65	9	

PSA: Prostate specific antigen: Single nucleotide polymorphism.

### Multi-factor analysis and the interaction analysis results

Multi-factor unconditional logistic regression analysis was carried out on potential PCa-related factors such as consumpltion of alcohol and tea, and the genotype of rs731236 and rs2735839. The results confirm that tea-drinking reduces the chance of PCa occurance (OR =0.58, 95% CI, 0.35 ~ 0.96). The genotype of Rs2735839 is related to the occurrence of PCa (P = 0.000), The chance of Pca occurance is 3.7 times higher for Rs2735839 carrying AG or GG factor compared to that carrying the AA (OR = 3.71, 95% CI = 2.02 to 6.83), as confirm in Table 5.

**Table 5:** Multivariate regression analysis of factors independently associated with prostate cancer

**Variable**	***β***	***X***^***2***^	***P***	**OR(95%/CI)**
Alcohol	0.42	1.185	0.276	0.85(0.64-1.14)
Tea	-0.56	4.44	0.035	0.58(0.35-0.96)
rs2735839	1.28	17.80	0.000	3.71(2.02-6.83)
rs731236	0.37	0.35	0.554	1.26(0.59-2.71)

PSA: Prostate specific antigen.

In order to further explore the interaction between the relevant factors of PCa occurrence, multiplying and additive model interaction analysis was used. The results of the adding the interaction analysis confirms no statistically significance of interaction between rs2735839 and tea consumption. Although no significant interaction between rs2735839 and tea consumption is found, within the group that has the the polymorphism loci rs2735839 KLK3 carrying the risk genotypes GG or GA, tea drinkers have a 55.1% reduction of the chance to have Pca, as seen in Table 6. In the Logistic regression analysis, the interaction term between environmental exposures-tea consumption and the KLK3 polymorphisms (SNPs) rs2735839 was included, and the results confirm that the interaction is statistically significant (P = 0.037).

**Table 6:** The interaction between the polymorphism of rs2735839 and environmental risk factors with PCa

**Genotype**	**Variable**	**Case (*n*=108)**	**Control (*n*=242)**	**OR 95% CI**	**Index RERI, 95% CI**
rs2735839	Tea				
AA	No	13	26	1.00	
AA	Yes	2	64	2.64 (056-12.53)	
GA+GG	No	55	36	13.72 (3.04-26.90)	-9.20 (-25.36-6.96)
GA+GG	Yes	38	116	6.16 (1.41-26.90)	

## Discussion

So far, the etiology and pathogenesis of PCa is still elusive. Nevertheless, the continuous development of immunology and molecular biology methods provide the basis for the diagnosis and prognosis of prostate cancer [12,13]. Lichtenstein et al [14] confirmed that 42% of the incidence of PCa can be attributed to genetic factors, and the rest is environment related. Studies on the relationship between the KLK3 and VDR polymorphisms as well as environmental factors and PCa occurance are very limited. Recent research on PCa whole-genome has revealed that several SNPs on chromosome 19q13.33 are related to the increased PCa risk [15]. The most significantly associated SNP is rs2735839, located at the downstream 600 bp of the KLK3 gene and its encoding is KLK3, known as PSA. In an independent research on the group genome, including PCa, lung cancer, colon cancer, and ovarian cancer, Thomas et al. [16] found no PCa-susceptibility on chromosome 19. Our research has confirm that, patients with the SNP of the KLK3 gene, rs2735839, carrying the allele GG and AG, has a PCa risk 3.7 times higher than that carrying A homozygotes (OR = 3.78, 95% CI = 2.03 ~~ 7.07, P = 0.00). Xu et al. [17] reported that in terms of the chance of carrying dangerous allele G in rs2735839 SNPs, of localized PCa patients was significantly higher than of the advanced PCa patients (P = 0.03). Gleason score growth and the risk of gene frequencies confirmed no statistical significance, which is consistent with our finding. Zheng et al. [18] confirmed based on the research on the Chinese population, that the SNPs rs2723839 polymorphism of KLK3 related with the risk of PCa; Kader et al. [19] confirmed that the frequency of the rs2735839 gene and Gleason score are statistically significant. The difference in the conclusions may be due to genetic susceptibility and racial factors, as well as sufficiency of the sample size.

Lundin et al. [20] first reported that vitamin D (VD) may be associated with the causes of PCa. Cell culture experiments confirmed that VD inhibits PCa [21]. Since VD produces physiological effects via its receptor (VDR), it is speculated that PCa susceptibility may depend on the polymorphism of the VDR genotype. The Taq I of several SNPs in VDR is located outside the 9th significant promoter region, which is SNPs rs731236. SNPs rs731236 and other flag material in this chromosomal region are stronly unbalanced; thus it is considered that this SNPs is strongly related to PCa occurance [22]. Medeiros [23] confirmed that in the VDR Taq I genes, rs731236 polymorphism, people who carry allele C homozygotes only have 1/3 (OR = 0.34, 95% CI, 0.16 ~ 0.76, P <0.01) of the risk to have PCa compared with those who carry miscellaneous zygote or T homozygous, therefore CC homozygotes gene is thought to prevent individual from suffering from PCa. However Gsur et al. [24] confirmed that within the Caucasian population in Austria, the VDR Taq I gene polymorphism is not related to the risk of suffering from PCa; the frequencies of the CC genotype in PCa patients and the corresponding controls, are 18% and 12%, respectively, and difference is not statistically significant (OR = 1.76, 95% CI, 0.90 to 3.45, P = 0 07). Our study confirms that within the Chinese Han population, the appearing frequencies of the genotype TT, TC, and CC in the SNPs, rs731236 (T/C) VDR of PCa patients and the control group are 88.89%, 9.26%, 1.85% and 90.50%, 9.10%, 0.40%, (P = 0.643), and correlation is found between rs731236 and the occurrence PCa. Our research indicates that The rs731236 polymorphism may not be associated with PCa occurrence among the Chinese Han population, combined with the previous research, it suggest that gene polymorphism is different between different races.

Tumorigenesis is affected by both environmental and genetic factors. Univariate analysis of the environmental factors confirmed that alcohol consumption and tea consumpiton are related to prostate illness, where alcohol consumption increases the risk of suffering from PCa while tea drinking is a protective factor. Multivariate Logistic regression analysis confirmed that tea drinking reduces the PCa occurance. Epidemiological studies have pointed out that the lower occruance rate of PCa among Asian population compared with the Westerners may be realted to the larger conumption of tea by the former [25,26]. The study has also found the correlation between the occurance of PCa and tea consumtion, which may be related to flavonols (protocatechuic) in green tea. Park [27] confirmed that protocatechuic can reduce PCa inplanted on mice. The analysis of environmental risk factors and genetic factors KLK3 gene polymorphisms (SNPs) rs2735839 revealed that multiplied interaction exists between tea consumption and rs2735839; Tea consumption may be against the dangerous gene GG + GA and protect people from PCa. Further in-depth research needs to be carry out in this aspect.

In summary, the results of this study suggest that the occurrence of PCa is related to the genotype of KLK3 SNPs rs2735839. The SNPs rs731236 genotype of VDR is not associated with PCa incidence. Environmental risk factors have impact on PCa occurance. Studies show that he expression of GOLPH3 and miR - 126 play a positive role in the malignant progression of PCa [28,29]. The relationship between the genetic, environmental factors and PCa incidence still need further in-depth investigation with a largeer sample size in order to provide a scientific basis for PCa prevention and control.

## Competing interests

The authors declare that they have no competing interests.

## Authors’ contributions

HJ carried out the molecular genetic studies, participated in the sequence alignment and drafted the manuscript. QZ carried out part the molecular genetic studies and Statistical analysis.

ZL charge of patient selection and specimen collection. CF charge for Subject design, research, data analysis and validation guidance papers writing. All authors read and final manuscript.
